# Patterns of maternal nutritional status based on mid upper arm circumference

**DOI:** 10.12669/pjms.36.3.1331

**Published:** 2020

**Authors:** Attia Bari, Nighat Sultana, Sana Mehreen, Nadia Sadaqat, Izza Imran, Rashida Javed

**Affiliations:** 1 Dr. Attia Bari, (DCH, MCPS, FCPS, MHPE), Associate Professor, Department of Pediatric Medicine, The Children’s Hospital and The ICH, Lahore, Pakistan; 2 Dr. Nighat Sultana, (FCPS), Assistant Professor, Department of Pediatric Medicine, The Children’s Hospital and The ICH, Lahore, Pakistan; 3 Dr. Sana Mehreen, (FCPS), Senior Registrar, Department of Pediatric Medicine, The Children’s Hospital and The ICH, Lahore, Pakistan; 4 Dr. Nadia Sadaqat, (FCPS), Senior Registrar, Department of Pediatric Medicine, The Children’s Hospital and The ICH, Lahore, Pakistan; 5 Dr. Izza Imran, (FCPS), Senior Registrar, Department of Pediatric Medicine, The Children’s Hospital and The ICH, Lahore, Pakistan; 6 Miss. Rashida Javed, (MSc Nutrition), Department of Pediatric Medicine, The Children’s Hospital and The ICH, Lahore, Pakistan

**Keywords:** Children, Malnutrition, Maternal nutrition, Mid upper arm circumference, WHZ score

## Abstract

**Objective:**

To determine the patterns of maternal nutrition status by using mid upper arm circumference (MUAC) and to examine the association of maternal nutritional status with the nutritional status of malnourished children under two years of age.

**Methods:**

Descriptive study conducted at the Department of Pediatric Medicine of the Children’s Hospital, Lahore from January 2017 to March 2018. A total of 227 mother accompanying their children admitted for nutritional rehabilitation were included. Demographics of participants along with MUAC of every mother was taken. Data analysis was done by SPSS 22.

**Results:**

Mean maternal age was 28.29±5.30 years and mean age of children was 9.22 ± 6.05 months. Mean maternal MUAC was 25.53±3.63 cm. Normal nutrition was present in only 70 (31%), 35 (15.4%) had moderate to severe under nutrition and 68 (30%) were overweight and 17 (7.5%) were obese. Maternal illiteracy was common 150 (66%) and 203 (89%) belong to poor social class. Majority 150 (75%) children had <-3SD WHZ score. Only 42 (18.5%) children were exclusively breast fed. Maternal malnutrition was significantly associated with severity of child’s undernutrition (p=0.045) and low rates of exclusive breast feeding practices (p=0.049).

**Conclusion:**

Malnutrition, in the form of both under nutrition and obesity is prevalent in mothers of malnourished children belonging to lower social class. Maternal illiteracy and low income are the major contributor in maternal malnutrition which in turn has an impact on child nutrition and breast feeding practices.

## INTRODUCTION

A large part of world’s population is affected by malnutrition, caused by inadequate, excess or imbalanced nutrition resulting in serious implications for individuals, communities and even to the country level. Maternal undernutrition is highly prevalent in resource-poor settings, ranging from 10% to 19%, but is particularly high (>20%) in sub-Saharan Africa and southeast Asia. In many developing countries, especially in South Asia maternal malnutrition in the form of macro and micronutrient deficiencies is a significant public health concern.^1^,^2^ Maternal mortality during birth process and overall disease burden for both mothers and their children is often due to maternal undernutrition, making it a special concern for health care providers and government as well.^3^,^4^ Malnutrition is recognized as a global issue and it is one of the key problems in Pakistani population especially targeting children and women.^5^

Mid-upper arm circumference (MUAC) is often used as a measure of fat-free mass. The gold standard for measuring body fatness is currently body mass index (BMI). For many decades MUAC has been used in children under the age of five to assess malnutrition. WHO defines severe acute malnutrition (SAM) by either MUAC <11.5 cm or weight for height Z score (WHZ) <−3SD or presence of edema.^6^ MUAC can be used as an alternative for BMI to determine under nutrition in older individuals and can be used to assess the risk of undernutrition in pregnant and lactating mothers.^7^

Researches in the adult population are available which have shown a strong correlation between MUAC and BMI. MUAC is considered to be a much simpler anthropometric measure as compared to BMI as it eliminates the need for height charts, accurate weighing scales and finally calculations required for BMI.^7^–^9^ An important advantage of using MUAC for nutritional assessment is that there is only minimal change in the MUAC during pregnancy, making it a better indicator of maternal nutrition during this period and afterwards as well.^9^ For screening women who are at risk for potential adverse pregnancy outcomes due to their poor nutritional status this simple method of MUAC measurement could reliably be substituted for BMI in the nutritional assessment in a resource limited settings.

MUAC can be used as an alternative to BMI, moreover MUAC is already being used in children to screen for malnutrition at a community level and now MUAC in adult population is increasingly being used as a surrogate marker to assess the nutritional status. There is scarcity of research on this topic from our country to analyze the women’s/ maternal health status of young children by measuring MUAC. The study aims were to determine the maternal nutrition status by using MUAC and to examine the association of maternal nutritional status with the severity of undernutrition in children under two years of age.

## METHODS

This was a descriptive cross sectional, conducted at The Children’s Hospital, Lahore from January 2017 to March 2018, on mothers admitted with their children 2 months to 2 years of age in the Stabilization center established by UNICEF for nutritional rehabilitation. After taking the approval from hospital Institutional Review Board (Ref. No: 2019-07-CHICH, dated January 1, 2019) and a well-informed written consent from the mothers data was collected and measurements were taken. Anthropometric measurements of MUAC was carried out on all mothers accompanying their children. The measurements taken for children were height and weight.

The MUAC was measured to the nearest 0.1 cm, midway between the acromion process of the shoulder joint and the olecranon of the elbow by using a standard flexible tape. The MUAC cut offs to classify nutritional status in mothers are: severe malnutrition <19 cm, moderate malnutrition ≥19 - < 22cm and mild ≥ 22 - 23cm, normal >23 - 30 cm and obese >30cm.^9^ A digital scale was used to measure the weight of the child with the child wearing only light clothes and recorded (to the nearest (0.1 kg). In children WHZ-score was taken to assess the nutritional status as compared to MUAC because children included were between 2 months to 2 years and MUAC is not recommended in children under 6 months of age. With an “infantometer” length of the child was measured (to the nearest 0.1 cm). These anthropometric measurements were converted to WHZ-scores. Malnutrition was categorized into severe acute malnutrition (WHZ score; <-3SD and moderate acute malnutrition (WHZ score; <-2SD). Data collection errors, was minimized by taking all measurements by our nutritionist and qualified and experienced staff nurse. Sample size of 227 was calculated by taking the prevalence of malnutrition in mothers as 18% in least developed countries with 95% confidence interval and 5% margin of error.^10^

Information about demographic profile, gender, breast feeding, top feeding type was noted. Their mother education, nutrition status was determined. Fig-1. Information about monthly income was also noted. Statistical software SPSS -22 was used for data collection. The quantitative variables like age and mid arm circumference was presented as mean and SD. Qualitative variables like gender, maternal education level and feeding practices was presented by calculating frequency and percentages. Chi-square test was used to find the association between the categorical variable and p-value of <0.05 was considered as significant.

## RESULTS

Mean maternal age was 28.29±5.30 years and majority 163 (72%) were in the age bracket of 20-30 years. Two third 150 (66%) were illiterate. Maternal socio-demographic characteristics are shown in (Table-I).

**Table-I:** Socio demographic characteristics of mothers.

Characteristics	Number	Percentage
*Maternal Age (mean 28.29 ± 5.30 years)*		
<20 years	3	1.3
20-25 years	81	35.7
26-30	82	36.1
31-40 years	60	26.4
>40 years	1	0.4
*Maternal Education*		
Primary	35	15.4
Secondary	31	13.7
Graduate	11	4.8
Illiterate	150	66.1
*Monthly Income*		
<10,000 PKR	21	9.4
10,000-25,000 PKR	182	80
26,000-50,000 PKR	24	10.6

Mean maternal MUAC was 25.53±3.63 cm, (n=36; 16%) had moderate to severe under nutrition and (n=17; 7%) were obese having MUAC >30 cm (Table-II).

**Table-II:** Demographic characteristics of children.

Maternal Nutritional Status	Percentage
Severe under-nutrition	2
Moderate under-nutrition	14
Mild under-nutrition	16
Normal weight	31
Over weight	30
Obese	7

Mean age of children was 9.22 ± 6.05 months. Majority children 150 (75%) were <-3SD WHZ-score having mean weight of 4.43±1.78 kg. Exclusive breast feeding was present only in 42 (18.5%) children (Table-III).

**Table-III:** Demographic characteristics of children.

Characteristics	Number	Percentage
*Child’s Age (mean 9.22 ± 6.05 months)*		
< 6 months	82	36.1
6 months - <1 year	65	28.6
1 year- 2 years	80	35.2
*Child’s feeding*		
Breast Fed (BF)	42	18.5
Top fed only	95	41.9
B.F & Top	90	39.6
*Child’s Nutritional Status WHZ-score*		
<-2 SD	57	25
<-3 SD	170	75

Significantly more mothers of children having WHZ-score <-3SD were severely and moderately undernourished (75%) as compared mothers of children having WHZ-score <-2SD (25%). Similarly, more mothers (62%, 76.5%) of children having WHZ-score <-3SD were overweight and obese respectively. Maternal malnutrition both in form of undernutrition and overweight and obesity was significantly associated with severity of child undernutrition (p=0.045). Failure to exclusive breast feed was associated with maternal undernutrition and obesity (p=0.049). There was no association of literacy and maternal age with the degree of malnutrition in children (p=>0.05).

## DISCUSSION

The Millennium Development Goal (MDG) of reducing maternal mortality by three-quarters (MDG 5) and now sustainable development goal of good health and well-being for all is highly related to women nutrition. Substantial number of cases of malnutrition in infancy and childhood are due to maternal malnutrition causing detrimental effects on both the mother and her child’s development.^11^ The rates of maternal malnutrition in the South Asian region are among the highest in the world. This is sometimes reflected as an overt malnutrition depicted by low BMI but majority mothers have subclinical micronutrient deficiencies.^2^ A study published in PJMS showed that maternal low level of hemoglobin, iron, cobalamin and folic acid are associated with these micronutrient deficiencies in the young children.^12^

The anthropometric measurements which are used to diagnose and monitor maternal malnutrition includes weight gain during pregnancy, BMI and MUAC.^13^ A review article narrated that WHO Collaborative Study has shown the importance of MUAC in identifying maternal malnutrition and MUAC cutoff values of <21 to 23 cm as having significant risk for LBW (OR 1.9, 95%CI: 95% 1.72.1).^14^ In our present study we analyzed the frequency of malnutrition; both under nutrition and over-weight or obesity in mothers of children admitted with moderate or severe malnutrition. We found that only one third mothers’ had normal nutritional status and one third were suffering from undernutrition and similar percentage were either overweight or obese. A study from South Africa had similar results of 20% under nutrition, 41% normal and 39% were over-weight or obese mothers and a study from Nigeria showing 31.8% mothers were malnourished.^9^,^15^ An Indian study also showed comparable results with one third of the study participants were underweight and about 18% were either overweight or obese.^16^ It high lights that both overweight and obesity are emerging problems in India. In resource-poor countries the type of food consumed mostly is carbohydrate leading to obesity trends in adults. Cereal consumption is the basic dietary ingredient in a research from Nepal and more than a quarter of the women were malnourished indicated by low BMI < 18.5 Kg/m^2^.^17^

A study on 4,981 women of reproductive age for nutritional status assessment both MUAC and BMI was used and it was found that MUAC can perform adequately in screening for under nutrition in women.^18^ Extensive literature searching revealed MUAC and BMI correlate with each other in assessing the nutritional status of adults and it was particularly researched for women of child bearing age. 7–9

Maternal education and social status plays a crucial role in the nutritional status of both women/mothers and their young children. Our two third mother were illiterate and our results are supported by a research from Pakistan in which 53% mothers were illiterate and this illiteracy had significant contribution in the nutritional status of child (p=0.01).^19^ Similarly a study from Ethiopia showed that 63% of women had no formal school education.^20^ It is not astonishing to see that illiteracy is prevalent in all resource poor countries as 58% mothers were illiterate shown in a study from Nigeria.^15^

Although lots of efforts are being put in for economic and social development but improving child nutrition is a daunting task and childhood malnutrition still remains a major public health concern in resource limited countries. In our study majority (75%) children had SAM with WHZ score of <-3SD. Most probable reasons of malnutrition in Pakistan are low income level, food insecurity and low food expenditures leading to low breast feeding.^5^ In our study only 18.5% of children were exclusively breast fed and failure to exclusive breast feed was significantly associated with maternal undernutrition and obesity (p=0.049). A study from china documented that the major reason for malnutrition and stunting in children is low rates of exclusive breastfeeding among children <6 months of age.^21^

In Pakistan now there is devolution of health to provinces with main focus on the health and nutrition of both children and women. The key to success and basic strategy for investing in the progress and in the interest of nation’s future is the implementation of a primary care strategy for women and children.^22^

### Limitations of the study

This was a single centered study and majority patients belonged to poor social class limiting the generalization of our results of other social class. Our results relied on only MUAC of mothers and we did not calculate the BMI of the mothers which may have given us more accurate picture of the nutritional status of the mothers. Secondly we included mothers of malnourished children only and children with normal nutrition were not included. We are now further extending our research by taking into account the BMI of mothers as well to see whether these results of MUAC are comparable with BMI and collecting maternal data from the children with normal nutrition and the results of this study will be published soon and the implication of this research is that MUAC can be used to assess maternal nutrition status and more focus should be given to both undernutrition and obesity.

## CONCLUSION

Malnutrition, in the form of both under nutrition and obesity is prevalent in our mother of moderate and severely undernourished children belonging to lower social class. Maternal illiteracy and low income are the major contributor in maternal malnutrition. This maternal malnutrition has an impact on child’s nutritional status and the failure to exclusive breast feed in young infants resulting in malnutrition in children.

### Author’s Contribution:

**AB:** Conceived, main author, manuscript writing and is responsible for integrity of research.

**NS:** Review, suggestions.

**SM:** Patient management.

**NS:** Data management.

**II:** Child’s Data collection.

**RJ:** Maternal data collection.
